# Enhanced Robustness of a Bridge-Type Rf-Mems Switch for Enabling Applications in 5G and 6G Communications

**DOI:** 10.3390/s22228893

**Published:** 2022-11-17

**Authors:** Jasmina Casals-Terré, Lluís Pradell, Julio César Heredia, Flavio Giacomozzi, Jacopo Iannacci, Adrián Contreras, Miquel Ribó

**Affiliations:** 1 Department Mechanical Engineering, Universitat Politècnica de Catalunya (UPC), C/Colom 7–11, 08222 Terrassa, Spain; 2 Department Signal Theory and Communications, Universitat Politècnica de Catalunya (UPC), C/Jordi Girona 1–3, 08034 Barcelona, Spain; 3 Fondazione Bruno Kessler-FBK, Via Sommarive 18, 38123 Trento, Italy; 4 Qorvo Inc., 1818 S Orange Blossom Trail, Apopka, FL 32703, USA; 5 Electronics and Telecommunications Department, La Salle-Ramon Llull University (URL), C/Sant Joan de La Salle, 42 08022 Barcelona, Spain

**Keywords:** RF-MEMS switch, beam suspension, coplanar-waveguide

## Abstract

In this paper, new suspended-membrane double-ohmic-contact RF-MEMS switch configurations are proposed. Double-diagonal (DDG) beam suspensions, with either two or three anchoring points, are designed and optimized to minimize membrane deformation due to residual fabrication stresses, thus exhibiting smaller mechanical deformation and a higher stiffness with more release force than previously designed single diagonal beam suspensions. The two-anchor DDGs are designed in two different orientations, in-line and 90°-rotated. The membrane may include a window to minimize the coupling to the lower electrode. The devices are integrated in a coplanar-waveguide transmission structure and fabricated using an eight-mask surface-micro-machining process on high-resistivity silicon, with dielectric-free actuation electrodes, and including glass protective caps. The RF-MEMS switch behavior is assessed from measurements of the device S parameters in ON and OFF states. The fabricated devices feature a measured pull-in voltage of 76.5 V/60 V for the windowed/not-windowed two-anchor DDG membranes, and 54 V/49.5 V for the windowed/not-windowed three-anchor DDG membranes, with a good agreement with mechanical 3D simulations. The measured ON-state insertion loss is better than 0.7 dB/0.8 dB and the isolation in the OFF state is better than 40 dB/31 dB up to 20 GHz for the in-line/90°-rotated devices, also in good agreement with 2.5D electromagnetic simulations.

## 1. Introduction

The development of microfabrication technologies, and in particular micro-electro-mechanical systems (MEMS), has a unique potential as a key enabling technology for all countries to develop the 6th generation of mobile communications and support the current implantation of the 5th generation (5G), and also to further enhance their space programs [1]. Currently, space programs are focusing on launching nanosatellites, which combine low cost and weight. Therefore, the miniaturization potential of MEMS devices and their reduced size and weight is an advantage for nanosatellites, and also for applications linked to 5G such as the Internet of Things [2,3], mobile and broadcasting communications [4,5,6], and wearable medical devices [7,8]. Researchers are working on the development of switches [4,5,6], phase shifters [9,10], switched antennas [11,12], filters [13,14,15] and amplifiers [16] all based on RF-MEMS switches as an alternative technology to conventional PIN diodes and transistor-based switches. However, the voltage required to mechanically actuate the MEMS switch (pull-in voltage, or V_pull-in_) is a critical parameter and many research efforts have been devoted to optimizing its value [5,6,17]. The pull-in voltage should be low to minimize complexity in high-voltage generation circuits, but there is a trade-off with the stability of the switch because lower voltages mean more flexibility of the suspension and therefore a less robust device.

Intensive research work is also being made to understand and minimize the impact of residual stresses during the fabrication processes on the switch performance [5,6]. Most fabrication processes involve the deposition of layers of different materials at different temperatures which results in the generation of residual stresses due to differences between the coefficient of thermal expansion of the materials, which in turn modify the initial topography of the MEMS device. Accurate models of this initial topography are needed to forecast the real actuation voltages [17,18,19]. In [18] an isolation test was proposed to precisely measure the deformations undergone by an Au-cantilever-based RF-MEMS switch. This method allows for prediction of the RF-MEMS deformation during the different high-temperature processes. In [17], a finite-element-method (FEM) model was fitted to the experimentally-measured curvature of thin films to determine their residual stress and then used to predict the initial curvature of the designs. In [20], a novel design approach based on the response surface method statistical methodology was proposed and validated against FEM commercial models.

From a mechanical point of view, work has focused on designing mechanical suspensions and membranes that are more robust to residual stresses. Most RF-MEMS switches have either straight or meander-type suspensions. These two configurations bend due to residual stresses during the manufacturing process. In [21], introducing corrugations in their suspensions was proposed to reduce the influence of the fabrication-induced stress on the membrane deflection, thus achieving a reasonable pull-in voltage (V_pull-in_) of 36 V, with an isolation of 30 dB and insertion loss of 0.7 dB up to 4 GHz. The corrugation required multiple layers of materials to be manufactured. On the other hand, in [22], a change in the suspension itself was proposed, which was partially a semi-circle combined with a straight-wide beam; in this case, the device was quite robust to stress changes, but with a high actuation voltage, between 80 and 90 V. In [23], a single material suspension was also proposed, as in [22], but with a much simpler design consisting in two diagonal beams supporting the membrane; the thermal expansion on one side was partially compensated for the symmetric expansion on the other side and thus the design improved the thermal stability.

It has been pointed out that the diagonal-beam suspension [23,24] produced more reliable switches, even though it increased the spring constant. In a previous authors’ work [24], diagonal (DG)-beams and circular beams were introduced to support either cantilever or bridge-type RF-MEMS switches embedded in a coplanar waveguide (CPW) transmission-line structure. These two configurations improved the performance of the RF-switches, since after release the suspensions minimized their deformation due to residual stresses. However, the membranes were still bent in some parts, thus reducing the required effective voltage to actuate the device, and the spring constant was less than 15 N/m in most cases, thus reducing the switch reliability. In [25], a novel manufacturing process optimized for RF applications was used in phase-shifter designs, corroborating the idea that membrane structures are more robust than cantilever-type switches. In [26], a novel design of RF-MEMs capacitive switches, based on a membrane and 4 zig-zag-type suspensions, was introduced. These suspensions were designed to compensate residual thermal and mechanical stresses; however, their suspension size, comparable to that of the switch, may limit its integration in RF circuits.

This paper will focus on the analysis, design, and characterization of a novel RF-MEMS switch, which features a double diagonal shaped suspension (DDG) optimized to minimize the deformation caused by residual fabrication stresses, and therefore it results in more robust switches with enhanced RF behavior. The design is validated through finite element analysis and experimental characterization and compared to the single diagonal suspension (DG) formerly proposed in [24]. The proposed RF-MEMS switch consists of a membrane structure embedded in a CPW transmission line which, upon actuation, performs a double series ohmic contact that bridges the input and output sections of the CPW central strip. The CPW transmission structure is advantageous because the metal conductors are disposed on the same plane (planar structure), and thus RF-MEMS switches are easily embedded providing reconfiguration capabilities by controlling the two propagation modes of a CPW, the CPW even mode or the CPW odd mode. This control is performed by switching the RF-MEMS device between its ON and OFF states. In [24], a double ohmic contact shunt switch was proposed to control the CPW odd mode. In contrast, the double ohmic contact series switch proposed in this paper is specifically designed to control the propagation of the more usual CPW even mode, providing very high isolation in the OFF state (much improved with respect to the ohmic switches reported in [24]) and small insertion loss in the ON state.

The paper is organized as follows. The RF-MEMS switch concept and behavior are presented in Section 2. The switch mechanical model, the fabrication process, and the RF design and electromagnetic (EM) simulations follow in Section 3. In Section 4, different switches are experimentally characterized, the results are discussed, and the mechanical numerical results and EM simulation results are validated. The conclusions of this work are presented in Section 5.

## 2. RF-MEMS Switches for Controlling the CPW Even Mode

**Figure 1:**
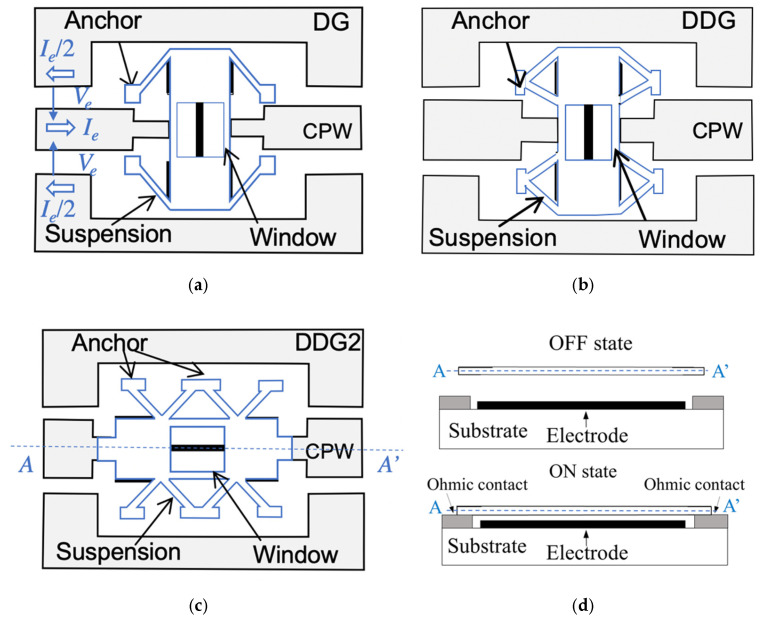
RF-MEMS switch suspension configurations. (**a**) Single diagonal beam (DG); (**b**) double diagonal beam (DDG); (**c**) double diagonal beam suspension with three anchoring points (DDG2). (**d**) Schematic device cross-section showing the membrane in the ON and OFF states.

**Figure 2:**
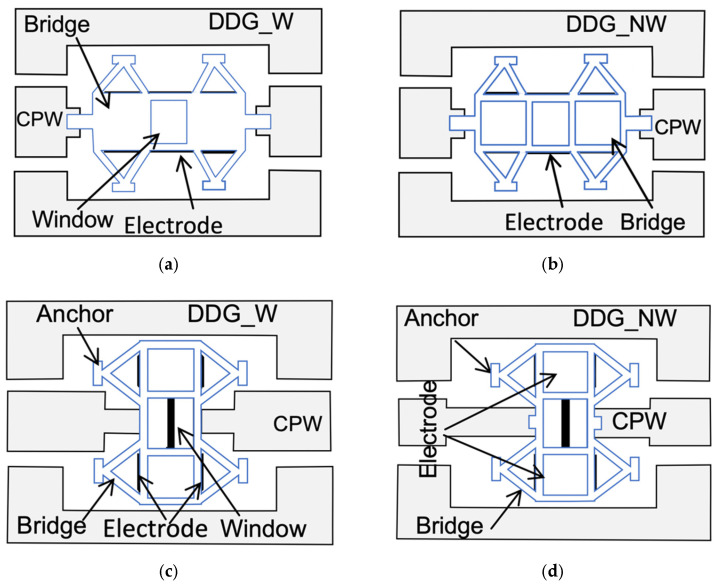
RF-MEMS switch membrane configurations and actuation electrodes for the DDG suspension. (**a**) DDG-W, in-line, (**b**) DDG-NW, in-line, (**c**) DDG-W, 90°-rotated, and (**d**) DDG-NW, 90°-rotated.

The proposed RF-MEMS switch suspensions are shown in Figure 1. They consist of an elevated metallic cantilever (movable membrane) anchored to the substrate and electrically separated from the remaining metal areas. The RF signal propagates along a CPW transmission line consisting of a central conductor and two side metal planes. The CPW even mode is defined by a voltage *V_e_* between the central conductor and the two side metal planes (which are at the same potential) and currents *I_e_* flowing on the central conductor and side planes in opposite directions (Figure 1a) [24]. The central conductor is interrupted beneath the MEMS membrane. Thus, when no actuation voltage is applied, the membrane is in its UP position and the CPW even-mode current flow is interrupted (switch in the OFF state). When a DC-actuation voltage equal or higher than the pull-in voltage is applied between the electrode and the movable membrane, the membrane collapses producing a double ohmic contact between the membrane and the two edges of the central conductor, thus allowing the CPW even-mode current flow through the switch (switch in the ON state). As shown in Figure 2, two possible membrane orientations have been considered, in-line (Figure 2a,b) and 90°-rotated (Figure 2c,d). While in in-line devices the double ohmic contact is performed between the narrow edges of the membrane and the central conductor, in 90°-rotated devices the double ohmic contact is performed between the wide edges of the membrane and the central conductor. As will be discussed in Section 3, the 90°-rotated device is expected to have a slightly lower ON-state series resistance than the in-line device, but at the expense of a higher OFF-state series capacitance. The membrane may have a window in the middle to minimize the unwanted capacitance between the bridge itself and the bottom electrode. The devices with a window in their membrane will be identified by the suffix “-W” (DG-W, DDG-W and DDG2-W), and those without a window will be identified by the suffix “-NW” (DG-NW, DDG-NW and DDG2-NW). In practice, this window has a negligible effect on the RF performance since the signal coupling in the OFF state is mainly due to a series capacitance which is a function of the distance between the two edges of the central conductor, as was observed from RF/microwave simulations (Section 3) and from experimental results (Section 4); its only effect was an increase in the pull-in/pull-out voltages.

**Figure 3:**
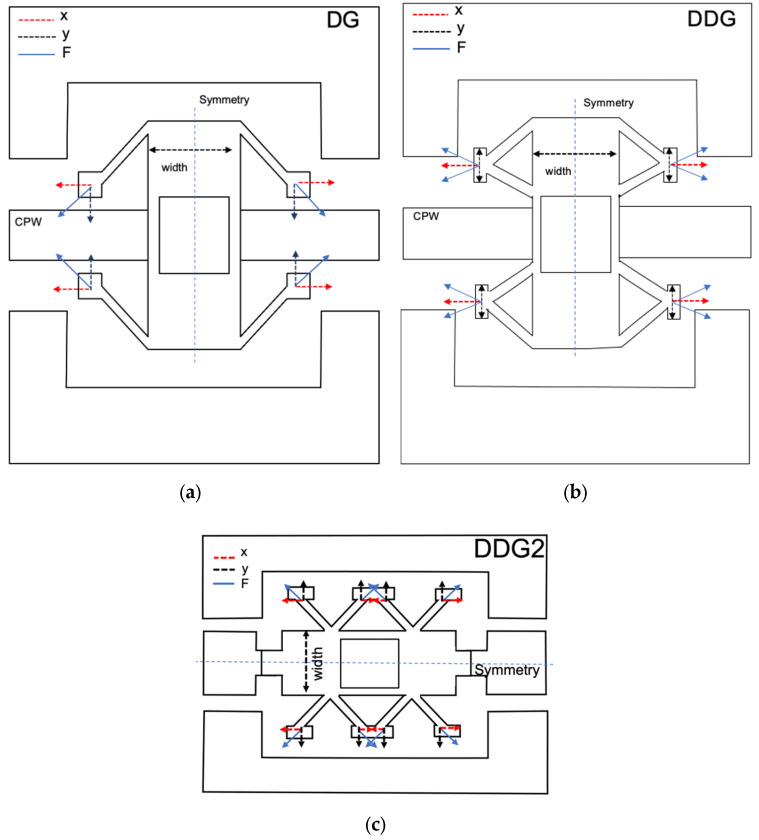
Comparison of new proposed mechanical suspensions (DDG, DDG2) with former DG suspension. (**a**) DG; (**b**) DGG; (**c**) DGG2.

During the deposition process of the different layers, residual stress forces are generated along the beam direction. Figure 3 shows the force direction of each suspension type. In the device with DG suspension, the forces compensate due to the symmetry of the device itself. However, this compensation occurs along the width and the length of the bridge, and small imperfections in the membrane can cause bending in DG devices, while in the DDG design the compensation along the length of the beam is not required because the suspension itself is symmetric and therefore the stresses produced in the suspension are cancelled by the suspension design. At the same time, the stiffness of the structure increases providing the switch with more release force. A modification of DDG with three anchoring points instead of two (DDG2) has also been considered; in this case some of the suspensions self-compensate (the middle ones) and some are compensated due to the symmetry of the device. It is therefore considered a combination of DG and DDG2 but with higher stiffness.

## 3. Materials and Methods

In this section the fabrication process for the switches proposed in the previous section is described, the FEM models obtained from ANSYS^®^ Workbench ^TM^ (Canonsburg, PA 15317, USA) and the RF design procedure using Keysight Momentum^TM^ 2.5D electromagnetic (EM) simulation (Keysight Technologies, Inc., Santa Rosa, CA 95403 USA) are discussed, and the fabricated switches are presented.

### 3.1. Fabrication Process

The technology used for the fabrication of the proposed RF-MEMS switches consists of an integrated eight-mask surface-micro-machining process, developed by the MEMS group of the FBK Institute [27,28].

**Figure 4:**
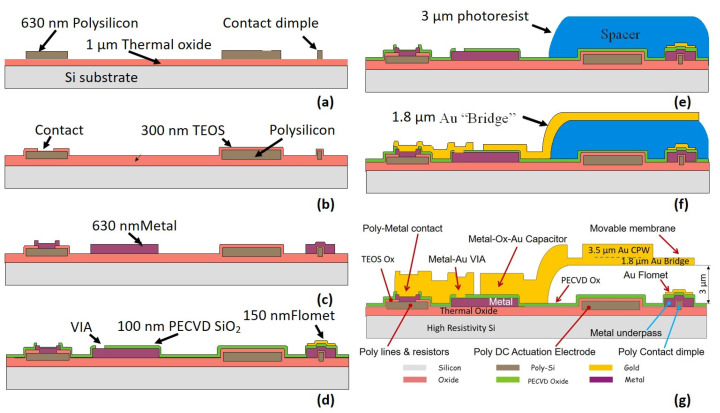
RF-MEMS switch fabrication-process flow Adapted with permission from Ref. [28]. 2022, Elsevier.

RF signal lines and ground areas are made of thick electroplated gold, to reduce insertion losses, while electrostatic-actuation electrodes and DC-bias signal lines are made of high-resistivity polysilicon, to minimize coupling with adjacent RF lines. The gold movable and suspended structures of the switches are electroplated over a sacrificial photoresist layer to define the required air gap. Two gold layers are used. A first thinner “Bridge” layer is used for flexible suspension legs and deformable parts while a second thicker “CPW” layer is superimposed to obtain the stiffer main body that moves rigidly, almost without deformations. The signal line under the suspended gold (metal underpass line) is realized in aluminium. On ohmic-contact switches, the gold-to-gold contact areas are defined by polysilicon protruding dimples underneath (Figure 4) to ensure a repeatable contact force and a uniform and reproducible low contact resistance.

A schematic of the fabrication process is reported on Figure 4. A one μm-thick silicon oxide isolation layer is obtained by wet oxidation of the 150 mm high-resistivity silicon wafers. The 630-nm-thick polysilicon layer actuation electrodes and corresponding signal lines are deposited by low-pressure chemical vapor deposition (LPCVD), lightly doped by B-ion implantation and defined by lithography and dry etching (Figure 4a). A 300 nm silicon dioxide insolation layer (TEOS) is deposited by LPCVD and the contact holes are defined by lithography and dry etched in the TEOS (Figure 4b). The 630 nm metal underpass lines are deposited by sputtering and patterned (Figure 4c). 100 nm-thick silicon oxide is deposited by PECVD to be used as metal isolation, as well as dielectric for capacitive contacts. To realize the vias and to remove the oxide over the dielectric-free actuation electrodes a selective dry etching is used. A thin film consisting of a 5 nm Cr adhesion layer and 150 nm of Au is then deposited by an electron-beam gun and patterned (Figure 4d) to realize electrically floating electrodes (Flomet) for capacitive contact switches and the bottom part of the gold-to-gold contacts for ohmic switches. To realize the air gap under the movable structures and suspended air bridges, a 3 μm-thick Fujifilm OIR305-20HC sacrificial photoresist (spacer) is deposed and lithographically defined (Figure 4e). The resist is then baked at 200 °C in order to be more stable and avoid deformations and damages during the next fabrication steps. A 2.5 nm Cr-25 nm Au seed layer is deposited all over the wafer and a thick AZ 4562 positive resist mold is defined. The 1.8-μm bridge gold layer is grown by electroplating inside the cavities (Figure 4f) carefully controlling the process parameters in order to obtain a slightly tensile residual stress [29]. The resist is removed, and a second AZ 4562 mold is defined for the selective electroplating of the thicker (3.5 µm) CPW gold layer superimposed to the first one to locally increase the bridge main-body rigidity. After resist removal, the Cr–Au seed layer is wet etched, and the electroplated gold is annealed at 190 °C to increase adhesion and slowly cooled to reduce internal stress. To release the movable and suspended structures the sacrificial resist spacer is then removed by oxygen plasma (Figure 4g). A lower process temperature results in a lower film stress and consequently lower bridge deformation but a much longer process time [30], therefore a tradeoff is required. Figure 4g also depicts a schematic cross-section of a capacitive switch (out-of-scale) showing the different layers and inter-layer contacts.

**Figure 5:**
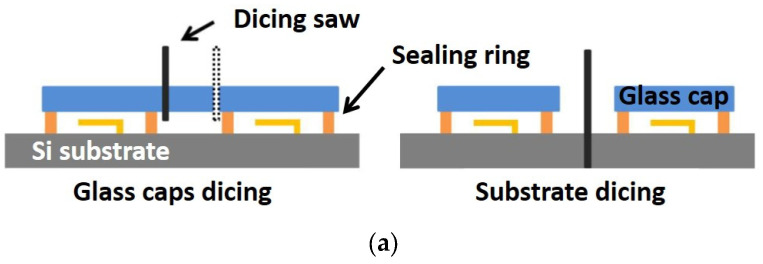

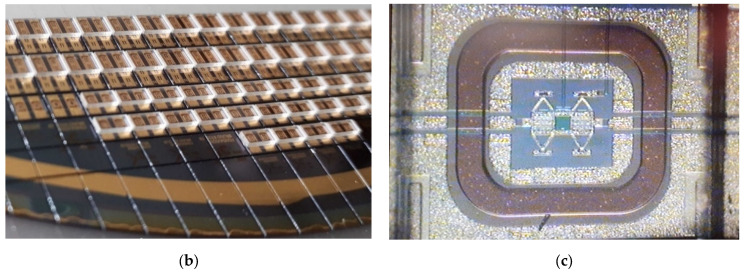
RF-MEMS switch dicing. (**a**) Dicing process schematic. (**b**) Picture of the fabricated wafer showing the RF-MEMS switches with wafer-level encapsulation; a single cap is used for every two switches. (**c**) Picture of a DDG-W fabricated device showing detail of CPW access lines, polysilicon bias lines, sealing ring and quartz cap.

To mechanically protect the delicate movable parts, glass caps with a polymer sealing ring can be applied to encapsulate the devices using either a wafer-to-wafer or a cap-to-die-bonding module [31,32]. The protective caps are realized using 150 mm-diameter, 500 µm-thick borosilicate glass with about the same thermal expansion coefficient as the silicon substrate [31,32]. A 50 µm-thick photosensitive dry film (ORDYL SY 355) is laminated at 105 °C and 1m/min over the glass wafers and patterned by lithography to obtain 100 µm-large sealing rings around the switches (Figure 5c). The glass wafers are then aligned over the silicon MEMS wafers and bonded in a nitrogen atmosphere to reduce moisture contamination. In the first step, a pressure of 3 MPa is applied at 100 °C for 30 min to allow the polymer to slightly deform, realize an intimate contact with the substrate and develop the bonding forces. After that, the full polymerization is obtained by increasing the temperature to 150 °C for a further 30 min, to improve adhesion and toughness. The bonding is not fully hermetic because moisture can slowly diffuse through the polymer, but it is robust enough to protect the MEMS from cooling water during the dicing process and from particulate contamination.

Dicing is performed in two steps. First only the quartz in excess over the pads and between contiguous dies is removed by a thin diamond blade adjusting the vertical blade position as shown on Figure 5a. In a second step the MEMS wafer is diced to separate the chips.

### 3.2. Mechanical Simulations

The previously described RF-switch manufacturing process induces intrinsic residual stresses due to the different deposition temperatures and different materials involved. 

This fact modifies the initially designed bridge and suspension shape and therefore changes the effective stiffness and increases the required actuation voltage. The ANSYS^®^ Workbench ^TM^ has been used to define the dimensions of different suspensions (see Table 1) using a coupled-field 3D FEM analysis to evaluate the design. The model takes into account the physical properties of the materials that are used during the manufacturing process of the switch (see Table 2 [24]). After meshing, the following boundary conditions are applied: the anchors are considered fixed supports. The analysis is run in two steps: initially, the loads are the residual stresses, which were taken from the measurements reported in [33]: σ_2_ = 58 MPa in the gold layer with a thickness t_2_ = 1.8 µm and σ_1_ = 62 MPa in the gold layer with a thickness t_1_ = 3.5 µm.

The results from this first analysis are used as the initial geometry for a coupled-field study that uses EMTGEN macro to create an array of nonlinear, coupled, electromechanical transducer elements (TRANS126). Therefore, the electrode is modelled through this macro and placed at the corresponding distance. In this simulation, the electrode is considered to be at 2.7 µm under the bridge, according to the thickness of the spacer in the manufacturing process (Figure 4). Table 1 lists the geometrical dimensions of the structures presented in this paper, along with the mechanical parameters obtained from numerical simulation (actuation voltage (V_pull-in_), spring constant, and mechanical resonant frequency). In the devices without a central window on the membrane, the electrode area is slightly bigger, and this fact decreases the required V_pull-in_, and even though the mass has increased, this increase is then balanced by the increase in the electrode area and therefore the resonance frequency is quite similar.

### 3.3. Switch RF-Design and Simulations

**Figure 6:**
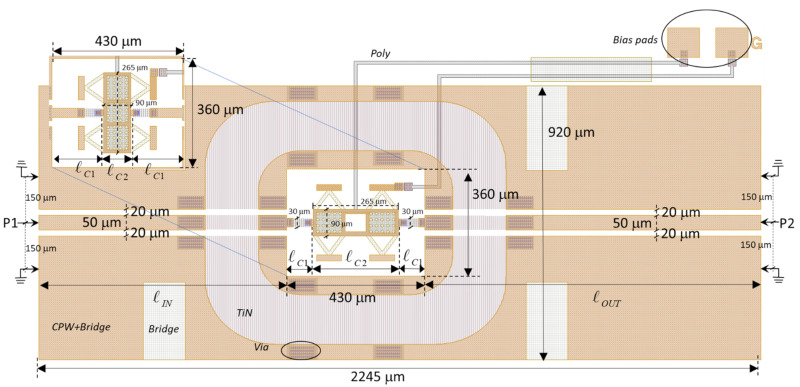
Layout of an RF-MEMS switch embedded in a planar rectangular cavity at a size of 430 μm × 360 μm defined on a CPW structure, showing the main dimensions (see Table 3 for ℓ_IN_, ℓ_OUT_, ℓ_C1_ and ℓ_C2_). P1 and P2 are the 50-Ω CPW even-mode input/output RF ports. A DDG-W in-line and a DDG-NW 90°-rotated (inset) configurations are shown as examples. The different layers are labeled according to Figure 4.

The RF/microwave design of the proposed RF-MEMS structures depicted in Figure 1 and Figure 2 was conceived as double-ohmic-contact switches able to control the signal propagation in CPW transmission structures [24]. This configuration was first proposed by the authors as a parallel switchable air bridge (SAB) with meander suspension to control the CPW odd mode [34], and it was later applied to microwave reconfigurable filters for Ku-band (12–18 GHz) [15]. In contrast to [15,34], the double-ohmic-contact switch proposed here is used in a series configuration and intended to control the CPW even mode instead of the CPW odd mode, and the membrane is suspended using DDG/DDG2 suspensions rather than meander ones. It has been designed to operate up to 20 GHz with high isolation (ISOL) in the OFF state (ISOL = −20 log|S_21_| with the membrane in the UP position) and very small insertion loss (IL) in the ON state (IL = −20 log|S_21_| with membrane actuated in the DOWN position). The switch membranes (all of them with the same size of 265 μm × 90 μm as shown in Table 1) are embedded in a planar rectangular cavity at a size of 430 μm × 360 μm defined on a CPW structure with dimensions shown in Figure 6, in which a DDG-W in-line switch and a DDG-NW 90°-rotated switch (inset) are shown as examples and fabricated on a 450 μm-thick high-resistivity silicon substrate (Hi-res Si) using the fabrication procedure discussed in Section 3.1.

The RF/microwave design was performed using Keysight Momentum^TM^ 2.5D electromagnetic (EM) simulation with the dimensions given in Table 1. The goal was to obtain the RF/microwave behavior of the two proposed orientations, in-line and 90°-rotated. While both of them have the same mechanical behavior, it is expected that the 90°-rotated configuration shows a slightly lower ON-state series resistance but worse isolation in the OFF state because the two edges of the central conductor are closer (100 μm) than in the in-line configuration (270.4 μm), thus allowing stronger signal coupling between the two edges of the CPW-central conductor. The EM simulation set-up considers the different dielectric and metal layers defined in Figure 4. The S parameters were simulated by considering the switch as a two-port device with ports defined for the CPW even-mode propagation as seen in the EM simulation layout shown in Figure 6.

**Figure 7:**
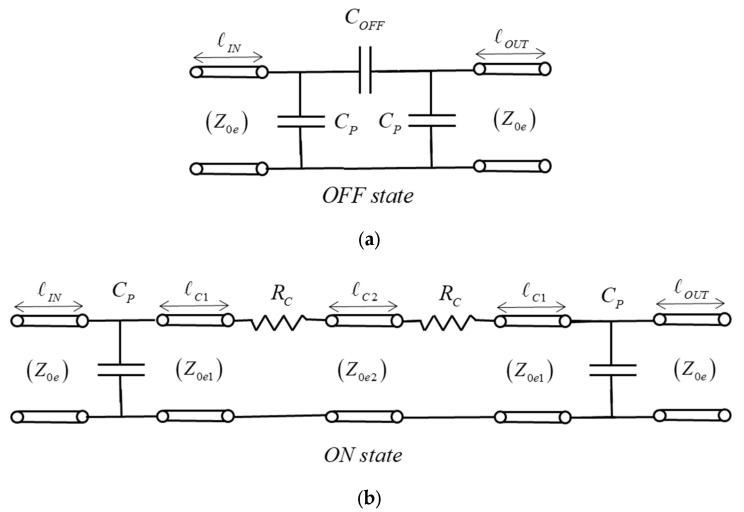
Equivalent circuits of the RF-MEMS series switch in the OFF (**a**) and ON (**b**) states. The lengths ℓ_IN,_ ℓ_OUT_ correspond to the input and output CPW transmission-line sections where the switch is embedded, as depicted in Figure 6. Z_0e_ = 50 Ω is the CPW even-mode characteristic impedance. Lengths ℓ_C1_ and ℓ_C2_ and characteristic impedances Z_0e1 and_ Z_0e2_ correspond to the cavity where the switch is embedded (see Table 3 for dimensions of ℓ_IN_, ℓ_OUT_, ℓ_C1_ and ℓ_C2_).

Equivalent circuits of the RF-MEMS switches were also proposed to ease the simulation of larger circuits in which the RF-MEMS might be embedded and for a better understanding of signal propagation in both states. Figure 7 shows the proposed equivalent-circuit topologies in the OFF and ON states. Its element values were obtained ex-post for a good agreement with the results of EM simulation. The switch basically behaves as a small series resistance in the ON state (R_ON_) due to the two ohmic-contact resistances (R_c_ in Figure 7b), and as a small series capacitance in the OFF state (C_OFF_). From previously designed DG switches with similar membrane dimensions [24], an ohmic-contact resistance of 1.5 Ω per contact (R_c_ = 1.5 Ω) can be anticipated, thus a value R_ON_ = 3 Ω can be expected. To the basic configurations in the ON state (R_ON_) and the OFF state (C_OFF_), CPW transmission-line sections (same lengths as in Figure 6) were added to the equivalent circuits, as well as to the small parallel parasitic capacitances (C_p_) that model the abrupt change in gap width between the CPW line and the planar rectangular cavity.

**Figure 8:**
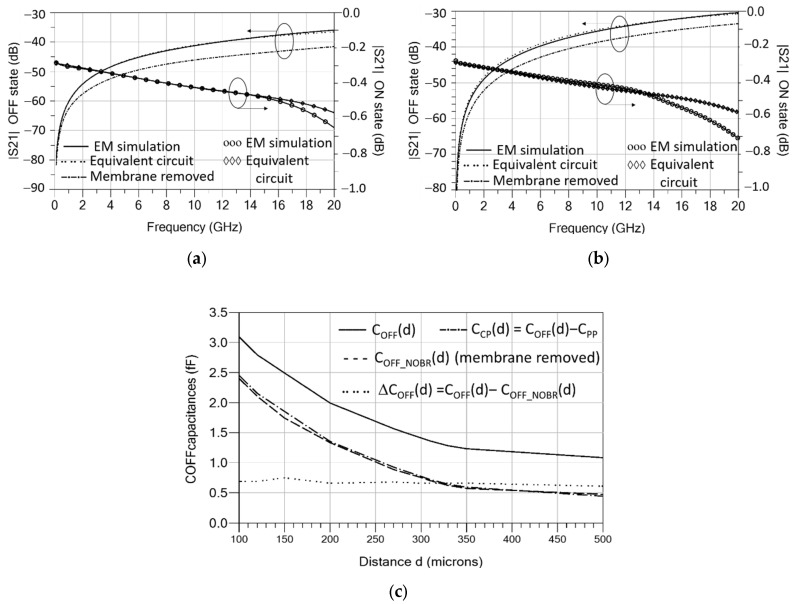
EM-simulated S_21_ parameter up to 20 GHz compared to the simulation of the equivalent circuits of Figure 7. (**a**) In-line configuration and (**b**) 90°-rotated configuration. (**c**) OFF-state capacitance fitted to EM simulations for different situations.

The EM-simulated transmission parameter (S_21_) up to 20 GHz of the circuit of Figure 6 in both states is plotted in Figure 8a,b, and compared to the simulation of the equivalent circuits shown in Figure 7. The two contact resistances R_c_ = 1.5 Ω were added in series to the results of Figure 8 in the ON state since the contact resistances are not included in the EM simulations. Observing the results of Figure 8, the in-line and 90°-rotated configurations show very similar behavior in the ON state, featuring a simulated IL < 0.7 dB and a good agreement with the equivalent circuit of Figure 7b. In the OFF state, the simulated isolation (ISOL) for in-line orientation (>36 dB) is higher than for the 90°-rotated orientation (>30 dB) as expected, and a good agreement with the equivalent circuit of Figure 7a is observed. In the OFF state, an EM simulation with the membrane removed was also performed and it is compared to the normal OFF-state simulation (with membrane). When the membrane is removed, a coupling can still be observed but at a lower level (−5 dB compared to the case with the membrane), thus showing a higher isolation.

To better understand the OFF-state behavior, the capacitance C_OFF_ is split into two contributions, a contribution due to signal coupling through a guided wave beneath the elevated membrane in the UP position between the two edges of the CPW-central conductor (C_CP_), and a contribution from the two parallel-plate capacitors arising between the elevated-metal contacts of the membrane in the UP position and the lower central conductor (C_PP_). The two contributions are in parallel, so that C_OFF_ = C_CP_ + C_PP_. Considering a membrane elevated 2.1 μm in the UP position above the TiN + FLOMET metal contacts (the air-gap height is reduced by 0.63-μm due to the polysilicon bumps in a part of the overlapping area, as shown in Figure 4) and a metal contact area of 266 μm^2^, this results in an approximate capacitance value C_PP_ = 0.64 fF. Regarding the signal coupling through the membrane in the UP position, modeled by C_CP_, this can only be predicted from EM simulation. To assess C_CP,_ a parametric study of the isolation (ISOL) as a function of the distance (d) between the two edges of the central conductor was performed using EM simulation, and the results were fitted to a distance-dependent capacitance C_OFF_(d). The distance d was swept from 100 μm (case of a 90°-rotated membrane) to 500 μm. Figure 8c plots four traces, C_OFF_(d), C_OFF_NOBR_(d) (C_OFF_ with the membrane removed), C_CP_(d) = C_OFF_(d)-C_PP_ and ΔC_OFF_(d) = C_OFF_(d)-C_OFF_NOBR_(d). It can be observed that C_OFF_ and C_OFF_NOBR_ decrease with distance, with them being strongly dependent on d between 100 μm and 350 μm. For values of d greater than 350 μm, the variation is much smoother. The range of values obtained is 3.09–1.08 fF (C_OFF_) and 2.40–0.47 fF (C_CP_). The coupling capacitance C_CP_(d) is almost the same as the capacitance in the case of membrane removed, C_OFF_NOBR_(d), as can also be seen by observing that ΔC_OFF_(d) remains mostly constant with distance to an approximate value of 0.69 fF, which is very close to C_PP_. For the nominal distances of the in-line and 90º-rotated devices (d = 270.4 μm/100 μm) the simulated C_OFF_ are: C_OFF_ = 1.56 fF (in-line) and C_OFF_ = 3.09 fF (90°–rotated). Table 3 summarizes the values of the equivalent-circuit elements fitted to the EM simulation. The switch cutoff frequency (f_CUTOFF_) is determined by C_OFF_. The cutoff frequency can be defined as the frequency at which the switch isolation ISOL (dB) is equal to a lower limit, and computed from the S_21_ parameter magnitude of a series capacitance C_OFF,_ $\left|S_{21}\right|=\frac{Z_{0}4\pi fC_{\mathrm{OFF}}}{\sqrt{1+{\left(Z_{0}4\pi fC_{\mathrm{OFF}}\right)}^{2}}}$, yielding

(1) $$f_{\mathrm{CUTOFF}}=\frac{1}{4\pi Z_{0}C_{\mathrm{OFF}}\sqrt{10^{\mathrm{ISOL}(\mathrm{dB})/10}-1}}$$

where *Z*_0_ is the reference impedance (*Z*_0_ = 50 Ω). The lower C_OFF_ is, the higher *f*_CUTOFF_ is. Using (1) and taking ISOL = 30 dB, it results in a theoretical upper frequency of *f*_CUTOFF_ = 32.2 GHz for the in-line devices and *f*_CUTOFF_ = 16.3 GHz for 90°-rotated devices. However, expression (1) is only an approximation to f_CUTOFF_ since it considers a series capacitance C_OFF_ only, but the equivalent circuit includes CPW line sections which also decrease the magnitude of S_21_ due to line loss. Taking all of these into account, the simulated f_CUTOFF_ values increase to 43.9 GHz and 22.3 GHz, respectively. Table 3 summarizes the values of the equivalent-circuit elements fitted to the EM simulation.

## 4. Experimental Results and Discussion

### 4.1. RF and Electrical Switch Characterization Methodology

**Figure 9:**
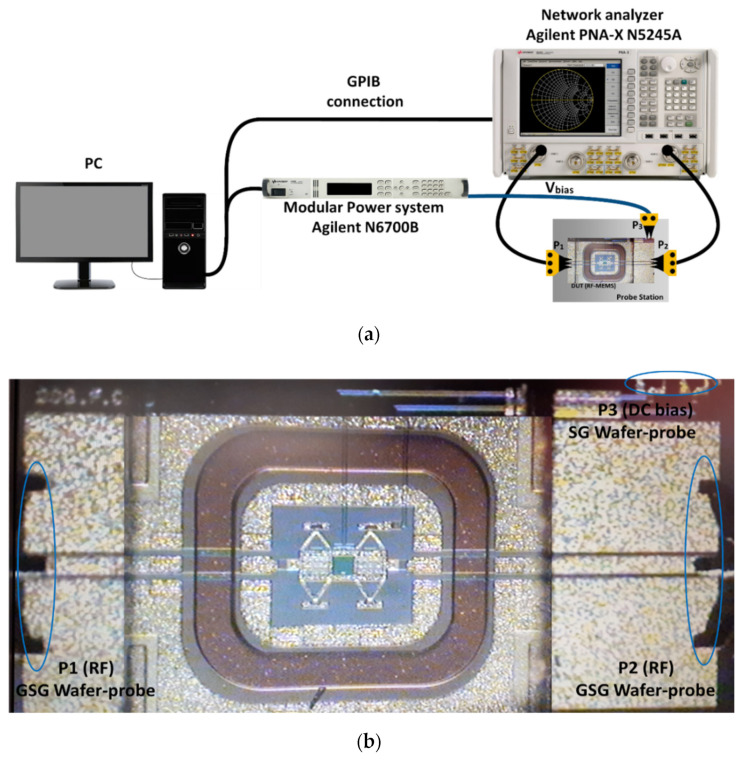
RF-MEMS switch characterization. (**a**) Experimental test bench for DC and RF characterization. (**b**) Picture of a measured RF-MEMS switch showing details of the GSG wafer probes for RF contacts and SG wafer probe for DC bias.

The straightforward characterization technique reported in [24] for both mechanical and RF/microwave behavior, based on the measurement of S parameters, was used: when the applied voltage increases and reaches the pull-in value there is an abrupt change in the magnitude of S_21_ from a very small value (isolation) to a value very close to 1 (0 dB insertion-loss), and the opposite occurs when the applied voltage decreases and the pull-out value is reached. The switch DC behavior (V_pull-in_ and V_pull-out_) was derived from the measurement of its transmission coefficient (S_21_ parameter) as a function of the applied voltage. Figure 9a shows the experimental set-up used for DC and RF characterization.

An Agilent N6700B power source supplied the DC bias. The S parameters were measured using an Agilent PNA-X N5245A network analyzer. The contact to RF-MEMS switches (Figure 9b) was performed using two 250 μm-pitch ground-signal-ground (GSG) wafer probes as RF ports (P1 and P2), and a third signal-ground (SG) wafer probe (P3) for DC-bias. 

### 4.2. Numerical Results of Mechanical Simulation and Comparison to Experimental Results

The three suspensions considered for the bridge type RF-MEMS switch are shown in Figure 1a–c and their dimensions are given in Table 1. The diagonal beam suspension DG has a V_pull-in_ of 28.5 V. If a window is added on the membrane, the V_pull-in_ increases due to a decrease in the area of actuation and becomes of 38.2 V. Similarly, for DDG-type suspensions the pull-in voltage is increased as obtained from mechanical simulation (Table 1). On the other hand, their stiffness is considerably increased, with it being 113.96 N/m (DDG-2 type) and 182.35 N/m (DDG type). The DDG2 type suspension interferes with the middle part of the CPW line, so in some applications this might be a handicap (they cannot be used in 90°-rotated configuration), but it has a lower V_pull-in_ compared to the DDG.

**Figure 10:**
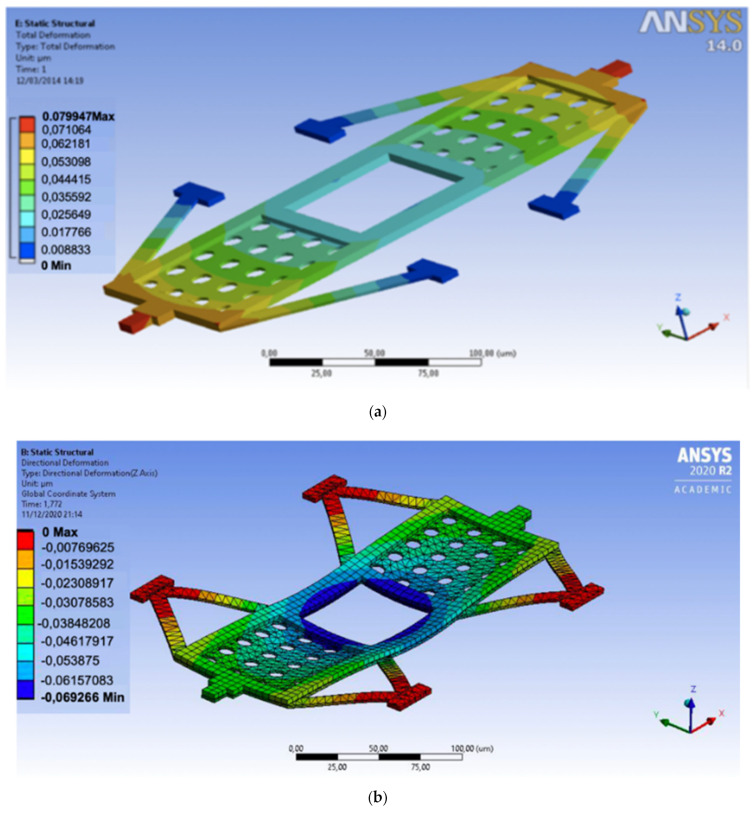
Initial deformation due to residual stress. (**a**) DG suspension; (**b**) DDG suspension.

The increased stiffness and the symmetry of the design minimizes the deformation due to residual stresses. DG and DDG have the same actuation area, but since the suspension of DDG tends to compensate the deformation along the length of the bridge, the contact point is only deflected due to the bending along the width of the bridge as shown in Figure 10. Figure 10a shows that suspensions are bended upward due to internal stress and DG contact point are 0.08 microns higher than the anchor point, while in the DDG case (Figure 10b), the deformation is smaller and the displacement between the anchor points and the contact point is only 0.03 μm and is favorable to actuation. DDG and DDG2 show a similar behavior in terms of residual stress compensation.

**Figure 11:**
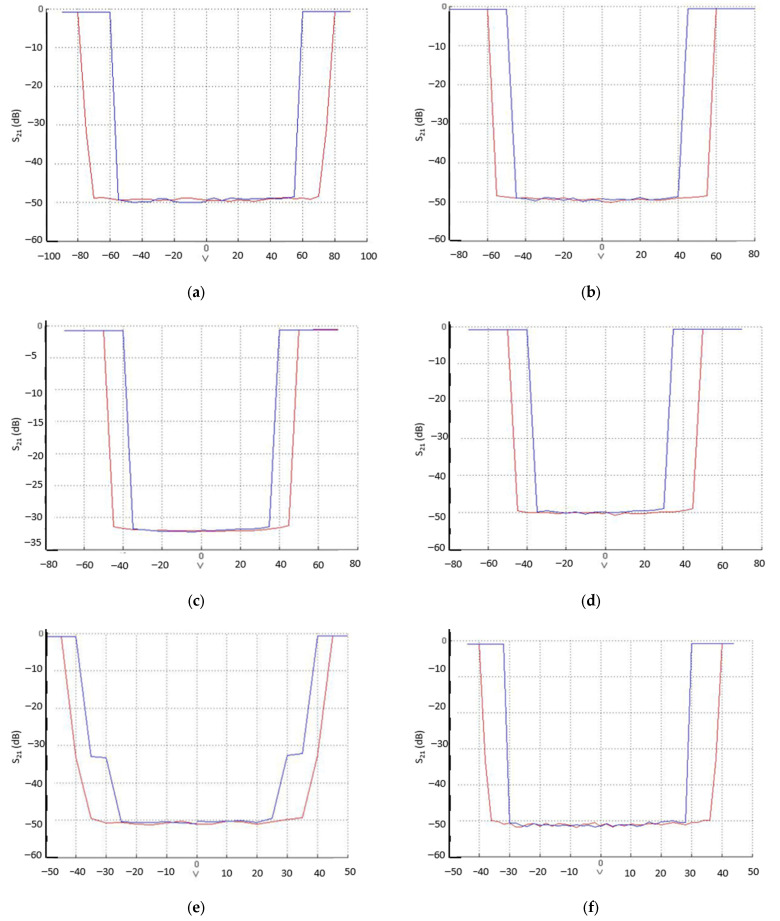
Hysteresis measurements of the fabricated series switches with two ohmic-contacts (g_0_ = 2.7 μm). Red trace: voltage variation from 0 V to ±V_pull-in_. Blue trace: voltage variation from ±V_pull-in_ to 0 V, showing ±V_pull-out_. (**a**) DDG-W-DL, (**b**) DDG-NW, (**c**) DDG2-W, (**d**) DDG2-NW, (**e**) DG-W, and (**f**) DG-NW.

Figure 11 shows the hysteresis measurements (magnitude of the transmission coefficient |S_21_|(dB) vs. actuation voltage) of the bridge-type switches shown in Figure 1, from which the actuation voltages (V_pull-in_) and the release voltage (V_pull-out_) can be easily inferred. The measurement frequency was 10 GHz. The measured values for V_pull-in_ are given in Table 4 and compared to the mechanical simulations in Table 1, and they show good agreement.

### 4.3. RF Experimental Results and Comparison to Results of EM Simulation

**Figure 12:**
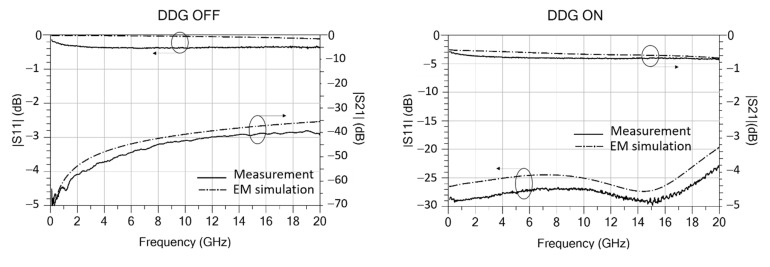

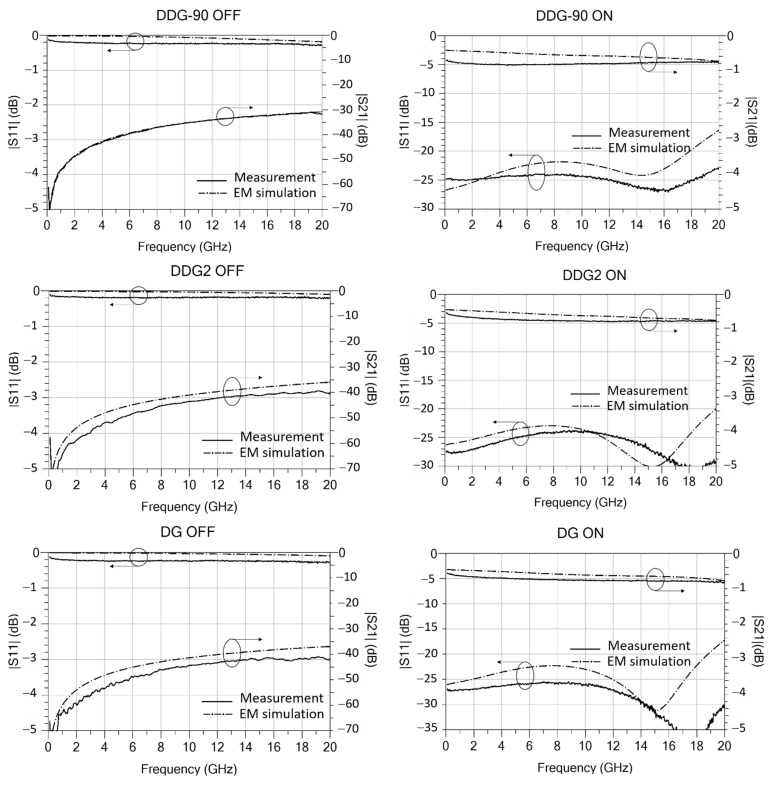
S-parameters of the fabricated RF-MEMS switches (Figure 1) measured using the set-up of Figure 9, compared to EM simulation. In-line configurations: DDG, DDG2, and DG. The 90°-rotated configuration: DDG-90.

The S-parameters of the different RF-MEMS switch mechanical configurations shown in Figure 1 were measured up to 20 GHz in both states (ON/OFF), using the set-up of Figure 9. The measurements are plotted in Figure 12 and compared to the EM simulations. Since windowed (-W) and not-windowed (-NW) configurations exhibit very similar RF performance they are not separately plotted. As predicted from simulations in Section 3.3, the 90°-rotated configuration shows smaller measured isolation in the OFF state. The measured isolation in the OFF state is greater than 40 dB for in-line switches (DDG-W/NW, DDG2-W/NW and DG-W/NW), and greater than 30 dB for 90°-rotated switches (DDG-90-W/NW); the measured insertion loss in the ON state is smaller than 0.72 dB (DDG-W/NW), 0.79 dB (DDG-90-W/NW and DDG2-W/NW), and 0.82 dB (DG-W/NW). The values of the equivalent-circuit elements in the ON and OFF states (Figure 8) were fitted to the measurements, and the results are listed in Table 5. The performance of switches in the ON and OFF states is also summarized in Table 5, where the different configurations are compared. The results show good agreement with simulations, thus validating the RF/microwave design methodology and circuit models proposed.

Table 6 compares the performance of the RF-MEMS switches proposed in this work with other RF-MEMS switches reported in the literature. Concerning the gap variation to uncompensated stress, the initial deformation is higher when the structure is a cantilever and the increase in the gap can achieve up to 9 μm [17]. When the RF-MEMS switch is double clamped in a membrane, this deformation decreases, with it achieving less than 0.03 μm in the current work. Concerning the RF performance, it can be noted that the proposed switch configurations feature a better isolation and a very similar (or slightly better) insertion loss than previous works at comparable frequencies.

## 5. Conclusions

In this paper, new membrane-suspension configurations for double-ohmic-contact RF-MEMS switches are proposed and analyzed. The suspensions are based on double-diagonal beams with either two (DDG) or three (DDG2) anchors, and the membranes may have a window to minimize coupling to the underneath actuation electrode. The two-anchor (DDG) device membrane has two possible orientations with respect to the signal propagation, in-line (DDG) or 90°-rotated (DDG-90). The proposed suspensions have been demonstrated to be robust to residual fabrication stresses, with them showing smaller mechanical deformation and higher stiffness with more release force than previously designed single diagonal beam (DG) suspensions. The switch membranes are embedded in a planar rectangular cavity at a size of 430 μm × 360 μm defined on a CPW structure; this way the CPW even-mode transmission through the series switch is controlled. Circuit models for the ON and OFF states have been derived to ease the simulation of larger circuits in which the RF-MEMS may be embedded and for a better understanding of signal propagation in both states. The OFF-state isolation has been studied in detail by EM simulation showing that the OFF-state series capacitance strongly depends on the distance between the two ohmic contacts, featuring 3 fF (DDG/DDG2) and 1.56 fF (DDG-90). The DDG, DDG2, and DDG-90 switches have been fabricated at FBK on high resistivity silicon, together with DG switches in order to compare performances using a well-proven eight-mask surface micro-machining process with dielectric-free actuation electrodes, and including glass protective caps. The simulated mechanical and RF/microwave performances, based on 3D-mechanical and 2.5D-EM simulations, respectively, have been validated through experimental measurements. Windowed and not-windowed configurations demonstrate different mechanical actuation voltages (76.5 V/60 V for DDG and 54 V/49.5 V for DDG2), but the same RF/microwave performance, featuring 0.7 dB/0.8 dB ON-state insertion loss and 40 dB/31 dB OFF-state isolation, up to 20 GHz, for the in-line-DDG and DDG-90 devices, respectively. The experimental results are in a good agreement with simulations, thus validating the RF-MEMS design and fabrication procedures.

## Figures and Tables

**Table 1:** Dimensions and numerical mechanical parameters.

Parameter	DG-W/NW	DDG-W/NW	DDG2-W/NW
Air gap (μm)	2.7	2.7	2.7
Supporting-beam width (μm)	10	11.2	8.25
Supporting-beam length (μm)	92	75.2	88
Angle (°)	30°	55°	45°
Bridge width (μm)	90	90	90
Bridge length (μm)	265	265	265
Window width	60/--	60/--	60/--
Window length	65/--	65/--	65/--
Bottom-electrodes area (μm^2^)	18,000/23,850	18,000/23,850	18,000/23,850
Contact area (μm^2^)	10 × 12.3	10 × 12.3	10 × 12.3
Spring constant (N/m)	39.9	182.35	113.96
Pull-in voltage (V)	38.2/28.5	81.7/70	65.6/56
Resonant frequency (kHz)	27.3	56.4	44.2

**Table 2:** Gold material properties Reproduced with permission from Ref. [24]. 2017, Springer Nature.

Symbol	Description	Value
σ	Residual stress (MPa) [33]	58–62
E	Young modulus (GPa) [33]	98.5 ± 6
ρ	Density (kg/m^3^)	19,840

**Table 3:** Equivalent circuit elements (Figure 8) and layout dimensions (Figure 6).

Parameter	In-Line Switch	90°-Rotated Switch
ℓ_IN_ (μm)	430.5	430.5
ℓ_OUT_ (μm)	705.5	705.5
ℓ_C1_ (μm)	79.8	165
ℓ_C2_ (μm)	270.4	100
C_p_ (fF)	5	5
Z_0e_ (Ω)	41.6	41.6
Z_0e1_ (Ω)	89.3	89.3
Z_0e2_ (Ω)	63.8	34.8
R_c_ (Ω)	1.5	1.5
C_OFF_ (fF)	1.56	3.09

**Table 4:** Measured actuation (V_pull-in_) and release (V_pull-out_) voltages from Figure 11 and simulated actuation voltages.

Parameter	V_pull-in_ (V)	V_pull-out_ (V)	V_pull-in_ (V) (Simulated)
DDG-W-DL	76.5 ± 1.5	57.5 ± 2.5	81.7
DDG-NW-DL	60 ± 0.5	46 ± 1.5	70
DDG2-W-DL	54 ± 4	40 ± 0.5	65.6
DDG2-NW-DL	49.5 ± 1.5	36 ± 2	56
DG-W-DL	40	30	38.2
DG-NW-DL	38	30	28.5

**Table 5:** Summarized measured performance at 20 GHz and equivalent-circuit elements for the different switch configurations.

Parameter	ISOL (dB)	IL (dB)	C_OFF_ (fF)	R_C_ (Ω)	C_P_ (fF)
DDG	40	0.72	1.06	2.1	5
DDG-90	31	0.78	3.09	2.1	5
DDG2	40	0.79	1.06	2.2	5
DG	42	0.81	0.96	2.2	5

**Table 6:** Comparison of the RF-MEMS switches proposed in this work with previous RF-MEMS switches reported in the literature.

Ref.	RF-MEMS Switch Structure	Stress (MPa)	V_pull-in_ (V)	Gap (μm)	Frequency (GHz)	Isolation (dB)	Insertion Loss (dB)
[5]	Cantilever	50	16	2	12.5	31	1.5
[6]	Cantilever	--	14.8	2.2	20	20	0.8
[17]	Cantilever	--	60	3 + 9 *	--	--	--
[19]	Membrane	100	60	2.6	40	15	1
[21]	Membrane	80	36	5	15	20	2.8
[22]	Membrane	100 + 4	90	0.25	40	14	0.8
[24]	Cantilever/ Membrane	58–62	10.6/38.2	2.7 + 0.08 *	15/15	10/10	0.9/0.9
**This work**	Membrane	58–62	65.6/56	2.7 + 0.03 *	20	40 (in-line)	0.7

* Increase in gap due to stress.

## Data Availability

Not applicable.
